# Oral Administration of Clinically Relevant Antimalarial Drugs Does Not Modify the Murine Gut Microbiota

**DOI:** 10.1038/s41598-019-48454-0

**Published:** 2019-08-16

**Authors:** Joshua E. Denny, Nathan W. Schmidt

**Affiliations:** 1 Department of Microbiology and Immunology, University of Louisville, Louisville, KY 40202 USA; 2 Present Address: Ryan White Center for Pediatric Infectious Diseases and Global Health, Department of Pediatrics, Indiana University School of Medicine, Indianapolis, IN 46202 USA

**Keywords:** Antiparasitic agents, Microbiome

## Abstract

Malaria is an infectious disease responsible for the death of around 450,000 people annually. As an effective vaccine against the parasite that causes malaria is not available, antimalarial drug treatments are critical in fighting the disease. Previous data has shown that the gut microbiota is important in modulating the severity of malaria. Although it is well appreciated that antibiotics substantially alter the gut microbiota, it is largely unknown how antimalarial drugs impact the gut microbiota. We show here that the two commonly used artemisinin combination therapies of artesunate plus amodiaquine and artemether plus lumefantrine do not change the gut microbiota. The overall relative species abundance and alpha diversity remained stable after treatment, while beta diversity analysis showed minimal changes due to drug treatment, which were transient and quickly returned to baseline. Additionally, treatment with antimalarial drugs did not change the kinetics of later *Plasmodium* infection. Taken together, antimalarial drug administration does not affect the gut microbiota.

## Introduction

*Plasmodium* is a significant global pathogen which infected 216 million people and caused 445,000 deaths in 2016; in sub-Saharan Africa, children under the age of 5 years old are the most vulnerable^1^. Currently, there is no effective vaccine against *Plasmodium*. Although there is growing resistance to frontline antimalarial drugs, particularly in Southeast Asia^2,3^, these drugs are still critical for treating those infected with *Plasmodium*. The gold standard for malaria treatment is artemisinin combination therapy (ACT). Artemisinin itself was originally isolated from the plant *Artemisia annua* in 1972 and has several semi-synthetic derivatives, including artemether, artesunate, and dihydroartemisinin^4^. Pharmacologically, these derivatives are more effective at clearing *Plasmodium* parasites than other anti-malarial drugs but tend to have shorter half-lives^5^. Consequently, artemisinin derivatives are usually combined with another anti-malarial drug that has a longer half-life to create an ACT to ensure continual parasite eradication and reduce instances of resistance to antimalarial drugs. Two of the most common ACTs are artemether combined with lumefantrine and artesunate combined with amodiaquine; these are commonly given orally but can also be administered intravenously, rectally, or intramuscularly^1^.

The mechanism of action for artemisinin and its derivatives has recently been shown to involve cellular damage in *Plasmodium*-infected red blood cells (iRBCs) along with inhibition of the proteasome; this leads to endoplasmic reticulum stress and the buildup of damaged proteins^6^. *In vitro* experiments have also shown that artesunate can generate reactive oxygen species within iRBCs that lead to caspase activation and apoptosis^7^. The mechanisms for lumefantrine and amodiaquine are currently unknown, but it is suspected that both drugs interfere with *Plasmodium*’s ability to detoxify heme^8^. Both types of mechanisms of action synergize to give ACTs their effectiveness in treating malaria.

Previous research has shown antibacterial effects of artemisinin and its derivatives on specific pathogens. Derivatives of artemisinin were shown to be differentially effective at killing various fungi and bacteria, including methicillin-resistant *Staphylococcus aureus* (MRSA)^9^. Additionally, encapsulation of artemisinin with beta-cyclodextrin enhances its antibacterial capacity when tested against MRSA^10^. While modifying artemisinin and its derivatives increases the antibacterial efficacy, these modified derivatives are not used clinically. However, unmodified artemether and artemisinin have been shown to be relatively potent at killing *Helicobacter pylori*, which is a human pathobiont^9,11^.

The gut microbiota has been shown to be important for many aspects of health and disease, including malaria. We have previously shown in mice that the gut microbiota can modulate the severity of infection, and modifying the microbiota through the diet was sufficient to make mice susceptible to severe malaria while antibiotic treatment followed by probiotics resulted in susceptible mice becoming resistant to severe malaria^12^. Additionally, *Plasmodium* infection itself can induce changes of the gut microbiota in mice during acute parasitemia that can result in long-term changes in the bacterial community composition, although these long-term changes did not affect susceptibility to future infection^13,14^. In contrast to what has been observed in murine models of malaria, a small study of Kenyan infants demonstrated that acute febrile *Plasmodium falciparum* infection was shown to have little effect on the composition of the stool microbiota; additionally, treating the infants with antimalarials after malaria diagnosis appeared to have no impact on the gut bacterial composition^15^.

Whereas oral antibiotic treatment has profound effects on the gut microbiota, the impact of antimalarial treatment on the gut microbiota is largely unknown. Using a mouse model, we show that two commonly used ACTs do not substantially change the gut microbiota at clinical dosing.

## Results

### Treatment with clinically relevant doses of acts does not change the taxonomic composition of the murine gut microbiota

**Figure 1:**
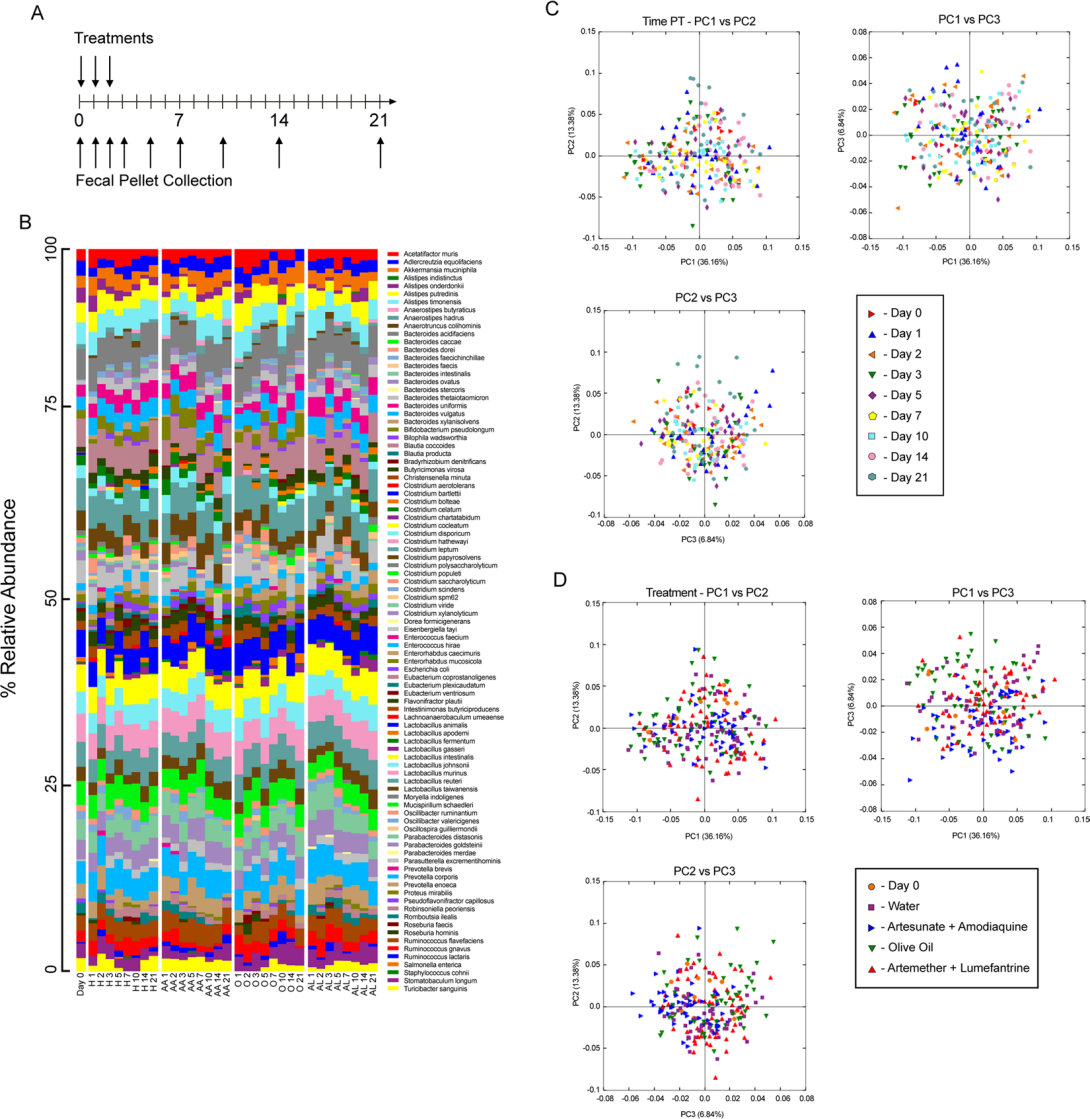
Relative bacterial abundances remain stable despite antimalarial treatments. (**A)** Experimental design for antimalarial treatments and fecal pellet collections. (**B)** Relative taxonomic abundances of bacterial species among different treatment groups. Time points are collapsed by average abundance and scaled to sum to 1. PCoA plots of weighted UniFrac distances between samples, with either (**C)** time p.t. or (**D)** treatment metadata distinguishing samples; this data is further analyzed in Figs 2 and 3. Data are the cumulative results of 2 experiments (4 mice/group/experiment); untreated (day 0) data for each group is pooled.

To test our hypothesis, we used two common ACTs: artesunate combined with amodiaquine (AA) and artemether combined with lumefantrine (AL). Due to the hydrophobic nature of lumefantrine, AL was dissolved in olive oil (O), while AA was dissolved in saline water (H). The ACTs and vehicle controls were given by oral gavage daily for three days, similar to clinical practice. Fecal pellets were collected before treatment (day 0) and at days 1, 2, 3, 5, 7, 10, 14, and 21 post-treatment (p.t.) (Fig. 1A) for DNA isolation and sequencing; replicates from the two experiments were pooled and all untreated samples were pooled as “day 0”.

Overall, the relative abundance of bacterial species remains consistent over treatments when compared to the pooled, pretreatment day 0 samples (Fig. 1B). When the weighted UniFrac beta diversity is viewed in a PCoA plot and the samples are colored by time p.t., there is no appreciable separation between any of the time points, indicating high similarity (Fig. 1C). Likewise, when the samples are colored by treatment received (Fig. 1D), the same trend is observed, with no appreciable clustering of samples, indicating neither ACT nor vehicle treatments lead to major changes. The beta diversity displayed here in Fig. 1C,D is further analyzed in the next section. Overall, when comparing the relative taxonomic abundances and beta diversity plots, there are no consistent patterns indicating changes due to antimalarial treatment.

### Antimalarial treatments do not affect the overall diversity of the murine gut microbiota

**Figure 2:**
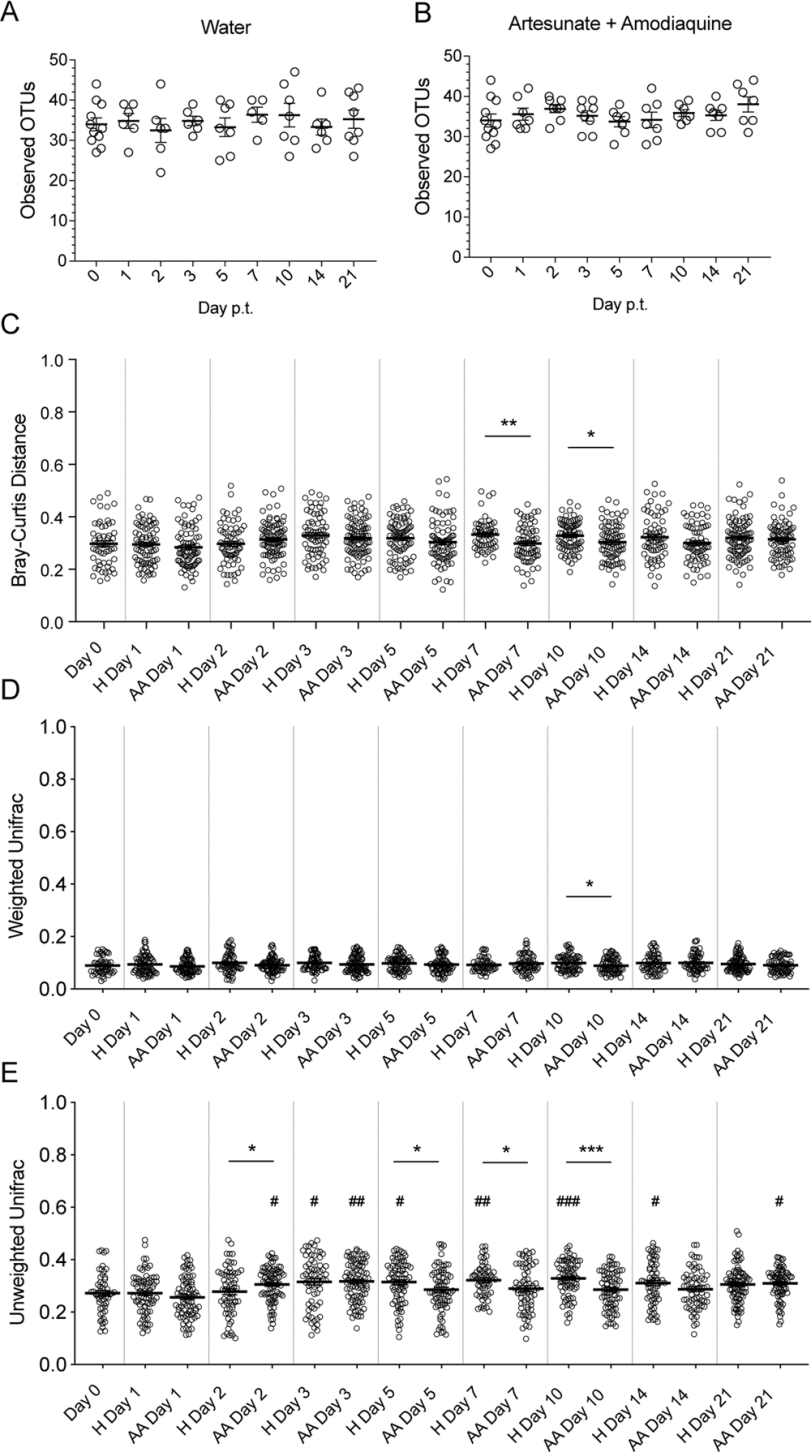
Antimalarial drug treatments cause minor changes to the diversity indices of the gut microbiota in mice treated with AA. Alpha diversity of the gut microbiota as measured by observed OTUs in mice treated with (**A)** saline water and (**B)** artesunate and amodiaquine. (**C)** Bray-Curtis beta diversity for samples treated with either water or artesunate and amodiaquine. The distances compared are between the pooled day 0 samples and each treatment group at each time point followed by comparisons between treatments at each time point. (**D)** Weighted UniFrac diversity for samples treated with either saline or artesunate and amodiaquine. (**E)** Unweighted UniFrac diversity for samples treated with either saline or artesunate and amodiaquine. Data (mean±SEM) in A-B were analyzed by one-way ANOVA with Tukey’s multiple comparisons test but showed no significant values (p > 0.05); data in (**C**–**E**) were analyzed by one-way ANOVA with Dunnett’s multiple comparisons test followed by a two-tailed t test for treatment comparisons at each time point. * = comparison between same-day treatments; ^#^ = comparison between treatment day and day 0 p.t. 1 symbol = p < 0.05; 2 symbols = p < 0.01; 3 symbols = p < 0.001; 4 symbols = p < 0.0001.

Alpha diversity analysis (measured by the number of observed OTUs per sample) of samples from mice treated with AA and the vehicle control, H, shows that the species composition does not change significantly (p > 0.05) over the course of the experiment (Fig. 2A,B). A two-step comparison was necessary for the beta diversity/dissimilarity comparisons to allow for both comparisons over time for the vehicle and treatment groups as well as comparisons between the vehicle and treatment groups on the indicated days p.i. First, the day 0 p.t. within-group dissimilarity is compared to either the vehicle-treated mice or the ACT-treated mice at each time point, with higher beta diversity indicating greater dissimilarity. This comparison will identify time points in either the vehicle- or ACT-treated mice that become more or less similar to the pre-treatment day 0 bacterial communities following treatments, designated here as the “day 0 comparison”. Second, the day 0 comparisons are then compared between treatments at each time point. Therefore, if the ACT-treated group is significantly different from the day 0 group and significantly different from the vehicle-treated group, it can be concluded that there is an ACT-induced change in the microbiota composition. With this in mind, neither H nor AA caused the gut microbiota to change from the day 0 baseline when analyzed using Bray-Curtis dissimilarity or weighted UniFrac (Fig. 2C,D).

When using the unweighted UniFrac distances for beta diversity comparisons there were several days (days 2, 5, 7, and 10 p.t.) where H- and AA-treated groups were different (Fig. 2E). However, the only time point where changes to the sample distances could be ascribed to AA treatment is day 2 p.t., as there is a significant difference between AA and day 0 but not H and day 0 along with a significant increase in AA compared to H (Fig. 2E). Of note, by day 3 p.t., the H dissimilarity is not different from AA, and by day 5 p.t., the AA samples have returned to baseline (Fig. 2E). Thus, while the AA-treatment at day 2 p.t. resulted in significant differences between both the H-treated day 2 p.t. group and the day 0 samples, this effect is short-lived, even with ongoing AA-treatment, and the small difference in beta diversity (0.2718 at day 0 versus 0.3055 for AA day 2, p = 0.028) point to natural variation rather than an AA-induced effect. Similarly, statistically significant differences seen in the Bray-Curtis and unweighted UniFrac analyses between the treatment groups, but lacking significant day 0 comparisons, indicates that the differences in treatment comparisons, while significant, are not biologically relevant, since the differences are driven by an increase in the vehicle-treated groups. Moreover, that these differences were not until 4 to 7 days after the last H- and AA-treatment when analyzed using Bray-Curtis and weighted UniFrac distances, respectively, suggests that these differences are not attributable to treatments. Finally, the lack of consistent differences between the treatment groups between the three measurements of beta diversity further emphasizes that the few differences that were observed are not biologically relevant; this claim is further explored in Fig. 4.

**Figure 3:**
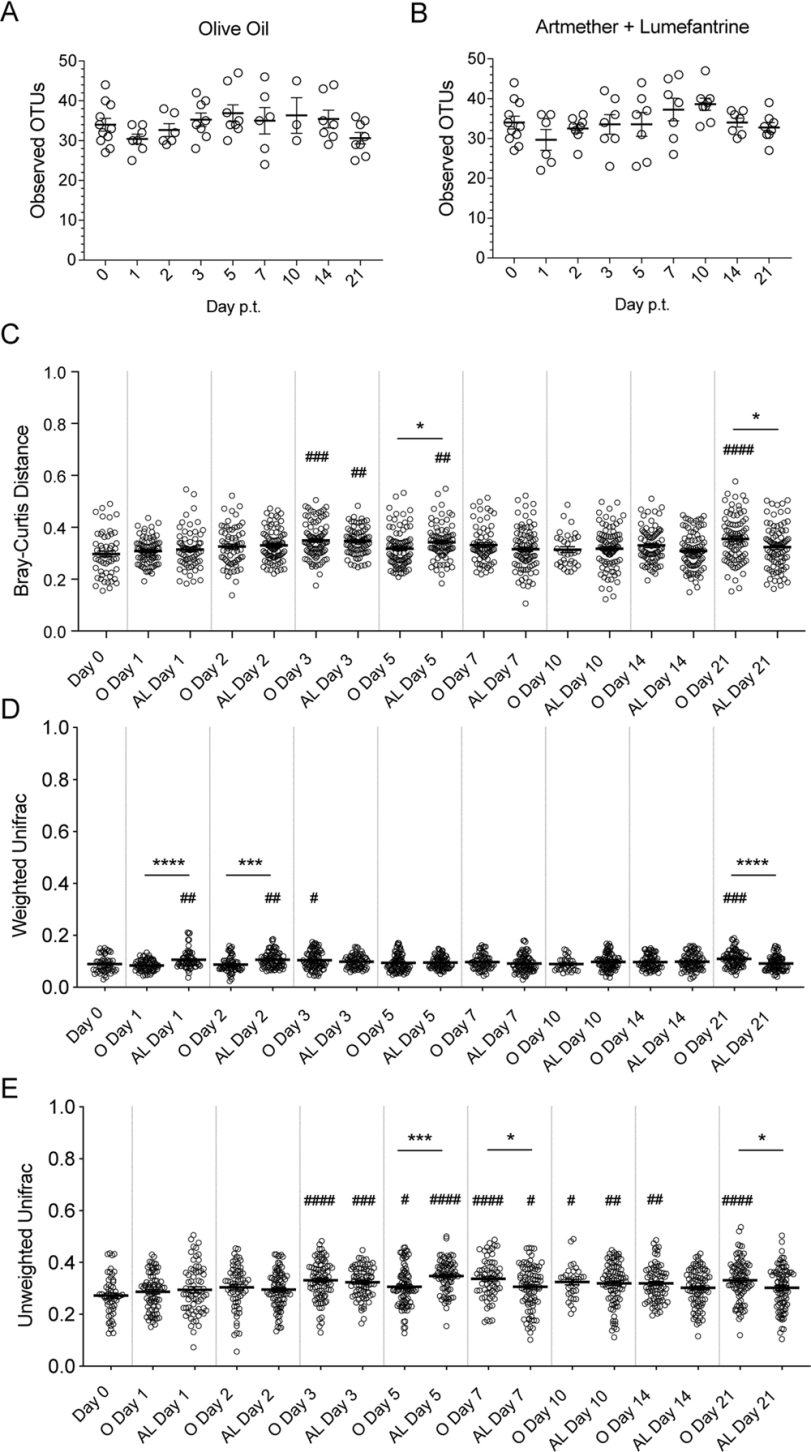
Antimalarial drug treatments cause minor changes to the diversity indices of the gut microbiota in mice treated with AL. Alpha diversity of the gut microbiota as measured by observed OTUs in mice treated with (**A)** olive oil and (**B)** artemether and lumefantrine. (**C)** Bray-Curtis beta diversity for samples treated with either olive oil or artemether and lumefantrine. The distances compared are between the pooled day 0 samples and each treatment group at each time point followed by comparisons between treatments at each time point. (**D)** Weighted UniFrac diversity for samples treated with olive oil or artemether and lumefantrine. (**E**) Unweighted UniFrac diversity for samples treated with olive oil or artemether and lumefantrine. Data (mean±SEM) in (**A**,**B**) were analyzed by one-way ANOVA with Tukey’s multiple comparisons test but showed no significant values (p > 0.05); data in C-E were analyzed by one-way ANOVA with Dunnett’s multiple comparisons test followed by a two-tailed t test for treatment comparisons at each time point. * = comparison between same-day treatments; # = comparison between treatment day and day 0 p.t. 1 symbol = p < 0.05; 2 symbols = p < 0.01; 3 symbols = p < 0.001; 4 symbols = p < 0.0001.

**Figure 4:**
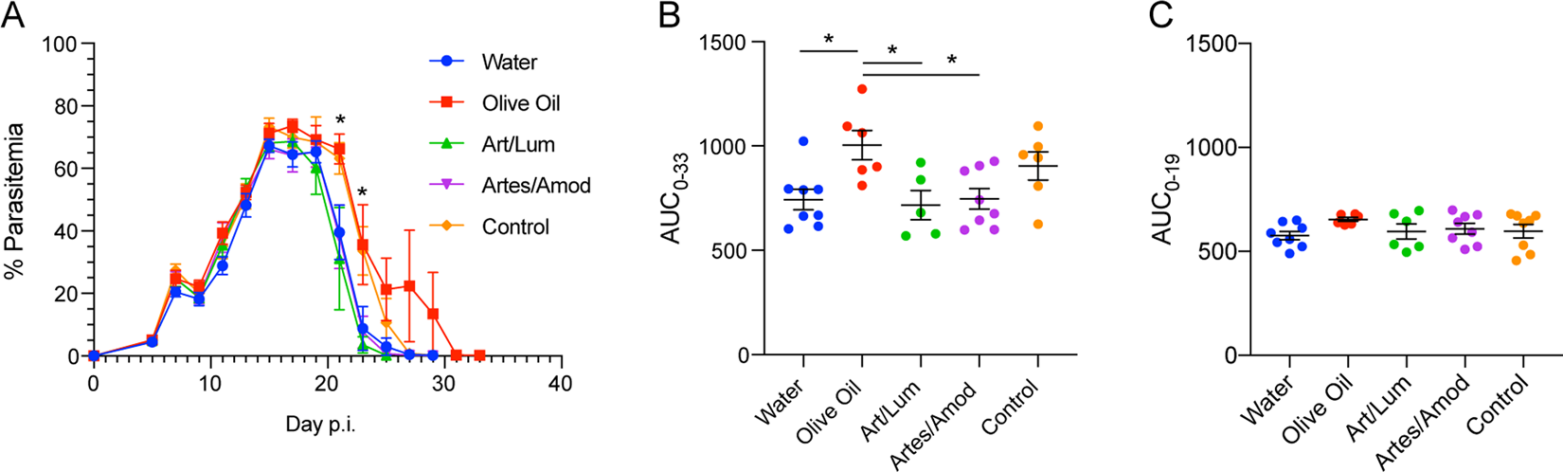
Antimalarial drug treatments do not change susceptibility to Py infection. Mice were treated with antimalarials followed by Py infection at 21 days p.t. (**A)** Percent parasitemia on the indicated day post infection (p.i.). (**B)** Area under the parasitemia curve (AUC) quantifying overall parasite burden (day 0–33 p.i.) during Py infection for each treatment group. (**C)** AUC quantifying parasite burden through peak infection (day 0–19 p.i.) for each treatment group. (**A–C)** Data (mean±S.E.) are the cumulative results of 2 experiments (4 mice/group/experiment). Data in (**A)** were analyzed by one-way ANOVA at each time point p.i.; *p < 0.05. Data in (**B)** were analyzed by one-way. ANOVA with Tukey’s multiple comparisons test; *p < 0.05.

For AL and its vehicle O, the alpha diversity for mice receiving either O or AL showed that there were no significant changes to the number of species found in the gut microbiota during the experiment (Fig. 3A,B). Using Bray-Curtis analysis there were only two time points, day 5 and 21 p.t., where there were significant differences between O- and AL-treated mice that also showed a significant day 0 comparison (Fig. 3C). As that these differences were not until 3 and 19 days, respectively, after the last O- and AL-treatment suggests that these differences are not attributable to treatments. Using weighted UniFrac, there were significant differences between O- and AL-treated mice at days 1, 2 and 21 along with O- or AL-treated mice also showing a difference between the day 0 samples (Fig. 3D). These data suggest that AL treatment may have altered the gut bacteria composition at days 1 and 2 p.t. Of note, there were minimal differences in the weighted UniFrac between these treatment groups (day 1, 0.1061 versus 0.0893; day 2, 0.1066 versus 0.0893). Finally, using unweighted UniFrac there were significant differences between O- and AL-treated mice at days 5, 7, and 21 along with O- or AL-treated mice also showing a difference between the day 0 samples (Fig. 3E). As noted above, that these differences were not until 2, 4 and 19 days after the last O- and AL-treatment suggests that these differences are not attributable to treatments. Moreover, since both groups are significantly different from day 0, the observed changes could also be attributed to the vehicle treatment. As with the H- and AA-treatments, the lack of consistent differences at any given time point between the treatment groups between the three measurements of beta diversity emphasizes that the few differences that were observed are not biologically relevant. The one exception is day 21 vehicle treatment that showed differences between all three beta diversity measurements. That this one consistent difference is 19 days after the last treatment, would suggest it is not likely attributed to the treatments.

Finally, the weighted UniFrac data was analyzed using the adonis function to identify differences in the composition of the microbiotas^16^. As adonis is sensitive to differences in sample dispersion, permdisp was used first to assess the homogeneity of variance of the samples. As seen in Table 1, the sample groups showed homogeneous variance. Analyzing the differences between the day 0, vehicle, and ACT treatment groups at each time point with adonis showed very few significant differences, only in the H- and AL-treated mice (Table 1). The R^2^ value for these comparisons was low, with the highest R^2^ value being 0.274, indicating that treatment is not a strong driver of compositional differences in these microbiotas. Unsurprisingly, the adonis analysis correlated well with our weighted UniFrac analysis in Fig. 3C, with days 1, 2, and 21 p.t. showing as significant in both analyses. The adonis analysis also picked day 14 p.t. in the H- and AL-treated mice as having differences in the microbiota compositions, which the analysis in Fig. 3C did not show; however, this time point is 12 days after the last AL treatment, meaning this difference is unlikely to be due to the antimalarial treatment. When these data and analyses are considered as a whole, there are several significant but minor differences in the microbiotas of mice treated with antimalarials, but no robust, long-term changes are seen that can be attributed to the antimalarial drugs.

**Table 1:** Comparison of the microbiota compositions across treatment groups at each time point.

	Permdisp		Adonis	
	Observed p	Permuted p	Adonis R^2^	Adonis p
AA1	0.0941	0.1	0.145	0.1
AA2	0.69	0.671	0.119	0.176
AA3	0.4989	0.485	0.065	0.593
AA5	0.2776	0.287	0.175	0.055
AA7	0.1437	0.122	0.217	0.096
AA10	0.3552	0.361	0.111	0.216
AA14	0.0786	0.061	0.245	0.036
AA21	0.9267	0.93	0.118	0.157
AL1	0.5292	0.532	0.189	0.02-*
AL2	0.607	0.598	0.274	0.003-**
AL3	0.1923	0.186	0.096	0.282
AL5	0.9849	0.988	0.108	0.215
AL7	0.8824	0.881	0.092	0.346
AL10	0.0555	0.087	0.188	0.152
AL14	0.1714	0.175	0.206	0.025-*
AL21	0.5362	0.531	0.159	0.04-*

Weighted UniFrac beta diversity data were analyzed using the permdisp and adonis functions to identify significant differences in microbiota composition. The adonis function was utilized at each time point to compare the vehicle, treatment, and day 0 groups. *p < 0.05; **p < 0.01.

### Infection with *Plasmodium yoelii* after antimalarial treatment shows no change in susceptibility to infection

To identify whether treatment with antimalarials changed the function of the gut microbiota and thus the severity of malaria despite not inducing long-term changes in the microbiota composition, mice were infected with *Plasmodium yoelii* 17XNL (Py) at day 21 p.t. (day 0 p.i.). Parasitemia was tracked and overall parasite burden was quantified by calculating the area under the parasitemia curve (AUC). Following infection, there were minimal differences in parasite kinetics between the treatment groups, with only two time points during resolution of infection where there was a significant difference in parasitemia between the groups (day 21, p = 0.0452; and day 23, p = 0.0229; Fig. 4A). When overall parasite burden is quantified using AUC, the only differences seen are between the O group and the other treatment groups (Fig. 4B); this is primarily driven by one O sample that took longer to clear the infection (Fig. 4A). Instead, when the AUC is quantified through the development and peak of infection at day 19 p.i., there are no significant differences between the treatment groups (Fig. 4C). Taken together, these data indicate that antimalarial treatments do not modify the microbiota and thus susceptibility to Py infection.

## Discussion

In this study, we have shown that treatment with common ACTs does not change the gut microbiota of mice or kinetics of later infection. This is an important observation, given the widespread use of these antimalarials around the globe and the diverse effect of the gut microbiota on the host, including its ability to shape the severity of malaria^12^. Importantly, this work needs to be corroborated in humans, as mice and the murine gut microbiota are not perfect analogs of humans. Of note, our recent work suggests that AL treatment does not impact the infant stool microbiome^16^.

Since the gut microbiota does not change appreciably, it does not appear that the antimalarial treatments affect the microbiota directly, despite previous research on the antibacterial effects of artemisinin and its derivatives^9,11^. However, in the context of an inflammatory disease like malaria that has been shown to have an effect on the composition of the gut microbiota^13–15^, antimalarials may in fact protect the microbiota. In a model of hepatitis, artesunate treatment inhibited production of inflammatory cytokines such as interferon gamma and tumor necrosis factor alpha while driving production of the anti-inflammatory IL-10^17^. The cytokine modulation was driven by inhibition of the NF- κB signaling pathway, as artesunate enhanced phosphorylation of the NF-κB pathway and signaling components in liver tissue^17^. In macrophages, artesunate has also been shown to block autophagy-dependent activation due to LPS and thus downstream production of inflammatory cytokines^18^. With this in mind, artemisinin and its derivatives may not only eliminate malaria parasites during infection but also have secondary effects in maintaining host gut homeostasis.

There is still much that is unknown about artemisinin and its interactions with *Plasmodium*. However, we have shown that two of the most commonly used ACTs do not change the composition of the gut microbiota in mice or alter the kinetics of *Plasmodium* infection. As the gut microbiota has become an important area of clinical investigation, it is beneficial to know that ACT treatments do not seem to pose the same challenges as antibiotic treatments to gut homeostasis and host health. However, given the differences between mice and humans, it is critical to continue characterizing the role of the human gut microbiota during malaria infection and treatment to enhance the effort to defeat malaria as a global pathogen.

## Methods

### Animals and housing

6-week old female C57BL/6N mice were purchased from Envigo (Indianapolis, IN). All mice were housed in a specific pathogen-free facility and acclimatized for a minimum of 7 days before starting experiments. Animals were fed the NIH-31 diet (Modified Open Formula Mouse/Rat Irradiated Diet; Harlan 7913; Envigo, Indianapolis, IN) and provided autoclaved, non-acidified reverse osmosis water ad libitum. The mice were kept on a 12-hour light/dark cycle from 6 AM to 6PM and 6PM to 6AM, respectively.

### Gut microbiota analysis

Mouse fecal pellets were collected at days 0, 1, 2, 3, 5, 7, 10, 14, and 21 p.t. and flash frozen in liquid nitrogen followed by storage at −80 °C. DNA was extracted using the QIAamp PowerFecal DNA kit (QIAGEN, Germantown, MD) according to the manufacturer’s instructions. DNA samples were then shipped overnight on ice to the Genome Technology Access Center at Washington University (GTAC, St. Louis, MO) for sequencing and analysis using the Multiple 16S Variable Region Species-Level IdentificatiON (MVRSION) algorithm^19^. The OTU table received from GTAC was CSS normalized and analyzed within QIIME v1.9.1 to produce taxa plots and calculate alpha diversity and beta diversity, specifically Bray-Curtis dissimilarity, unweighted UniFrac and weighted UniFrac^20,21^. The OTU table from GTAC and associated metadata map file are included as supplemental data files with this manuscript as Supplemental Tables S1 and S2, respectively. Forward and reverse read counts for each sample are included in Supplemental Table S3.

### Antimalarial drug treatments

For both AL and AA, clinical dosing was used according to recommendations by the World Health Organization^22^. Artemether was given at 2 mg/kg and lumefantrine at 12 mg/kg, while artesunate was given at 4 mg/kg and amodiaquine at 10 mg/kg. AL and AA were diluted in either 100% olive oil (Kroger, Cincinnati, OH, USA) or saline (0.9%, Teknova, Hollister, CA, USA), respectively. Mice received AL and AA by oral once daily for three days. There were 2 experiments total (4 mice/group/experiment).

### Plasmodium Infection

C57BL/6N mice were injected intravenously with 1 × 10^5^ red bloods cells (RBCs) infected with *Plasmodium yoelii* 17XNL diluted in 200 μL of saline, prepared from frozen stock. Parasitemia, the percentage of infected red blood cells, was monitored using flow cytometry beginning on day 5 p.i. and continuing every other day until clearance of the parasite using blood taken from the tails of infected mice. From each mouse, 5 μL of whole blood was diluted in 100 μL of cold PBS, fixed in 0.00625% glutaraldehyde, and then stained. The staining panel included CD45.2-APC (clone 104; Biolegend, San Diego, CA), Ter119-APC/Cy7 (clone TER-119; Biolegend, San Diego, CA), dihydroethidium (MilliporeSigma, St. Louis, MO), and Hoechst 33342 (MilliporeSigma, St. Louis, MO); samples were then resuspended in flow cytometry buffer and analyzed. Single cells were gated by Ter119^+^CD45.2^−^ followed by gating the infected subpopulation on dihydroethidium^+^Hoechst 33342^+^ to identify percent parasitemia. There were 2 experiments total (4 mice/group/experiment).

### Statistical analysis

Statistical analyses were performed using GraphPad Prism 7 software (GraphPad Software, La Jolla, CA, USA). For all analyses, the alpha was set at 0.05. For area under the curve (AUC) parasite burden analyses, the trapezoidal rule was used for Eq. (1):

1 $${{\rm{AUC}}}_{({\rm{t}}1-{\rm{t}}-{\rm{last}})}={\rm{\Sigma }}\,({{\rm{p}}}_{{\rm{i}}}+{{\rm{p}}}_{{\rm{i}}+{\rm{1}}})\ast ({{\rm{t}}}_{{\rm{i}}+{\rm{1}}}-{{\rm{t}}}_{{\rm{i}}})/2$$

where “p” is percent parasitemia at the designated time point “t”^23^.

Data in Figs 2A,B and 3A,B were analyzed by one-way ANOVA with Tukey’s multiple comparisons test; data in Figs 2C,D and 3C,D were analyzed by one-way ANOVA with Dunnett’s multiple comparisons test followed by a two-tailed t test for treatment comparisons at each time point. Data in Fig. 4B,C were analyzed by one-way ANOVA with Tukey’s post-hoc multiple comparison’s test between treatment groups.

Data in Table 1 were analyzed using the compare_categories.py function in QIIME, specifically the permdisp and adonis functions.

## Declarations

### Ethics approval

All animal experiments were carried out at the University of Louisville in compliance with local and national regulations of laboratory animal welfare. Additionally, all animal care and use procedures were approved by the University of Louisville Institutional Animal Care and Use Committee.

## Supplementary information

Supplemental Table 1

Supplemenetal Table 2

Supplemental Table 3

## Data Availability

All data generated or analyzed during this study are included in this published article and the supplementary information files. 16S rRNA gene sequences have been deposited in the NCBI Sequence Read Archive under the BioProject accession number PRJNA494178 located at https://www.ncbi.nlm.nih.gov/bioproject/PRJNA494178/. The OTU table from GTAC and associated metadata map file are included as supplemental data files with this manuscript as Supplemental Tables S1 and S2, respectively. Forward and reverse read counts for each sample are included in Supplemental Table S3.
